# Comparison of middle-term valve durability between transcatheter aortic valve implantation and surgical aortic valve replacement: an updated systematic review and meta-analysis of RCTs

**DOI:** 10.3389/fcvm.2023.1242608

**Published:** 2023-09-13

**Authors:** Tsahi T. Lerman, Amos Levi, Troels Højsgaard Jørgensen, Lars Søndergaard, Yeela Talmor-Barkan, Ran Kornowski

**Affiliations:** ^1^ Department of Internal Medicine F-Recanati, Rabin Medical Center, Beilinson Hospital, Petah Tikva, Israel; ^2^ Department of Cardiology, Rabin Medical Center, Petah Tikva, Israel; ^3^ The Faculty of Medicine, Tel Aviv University, Tel Aviv, Israel; ^4^ Department of Cardiology, The Heart Center, Rigshospitalet, Copenhagen University Hospital, Copenhagen, Denmark

**Keywords:** TAVI, SAVR, durability, structural valve deterioration, bioprosthetic valve failure

## Abstract

**Background:**

This study aims to compare valve durability between transcatheter aortic valve implantation (TAVI) and surgical aortic valve replacement (SAVR).

**Methods:**

We conducted a systematic review and meta-analysis using data from randomized controlled trials (RCTs). The primary outcome was structural valve deterioration (SVD). Secondary outcomes were bioprosthetic valve failure, reintervention, effective orifice area (EOA), mean pressure gradient, and moderate–severe aortic regurgitation (AR, transvalvular and/or paravalvular).

**Results:**

Twenty-five publications from seven RCTs consisting of 7,970 patients were included in the analysis with follow-up ranges of 2–8 years. No significant difference was found between the two groups with regard to SVD [odds ratio (OR) 0.72; 95% CI: 0.25–2.12]. The TAVI group was reported to exhibit a statistically significant higher risk of reintervention (OR 2.03; 95% CI: 1.34–3.05) and a moderate–severe AR (OR 6.54; 95% CI: 3.92–10.91) compared with the SAVR group. A trend toward lower mean pressure gradient in the TAVI group [(mean difference (MD) −1.61; 95% CI: −3.5 to 0.28)] and significant higher EOA (MD 0.20; 95% CI: 0.08–0.31) was noted.

**Conclusion:**

The present data indicate that TAVI provides a comparable risk of SVD with favorable hemodynamic profile compared with SAVR. However, the higher risk of significant AR and reintervention was demonstrated.

**Systematic Review Registration:**

PROSPERO (CRD42022363060).

## Introduction

Since its clinical introduction in 2002 by Dr. Alain Cribier, transcatheter aortic valve implantation (TAVI) has become a procedure of choice for treating severe symptomatic aortic stenosis (AS) in high-risk and elderly patients (1–3). Continuous research and development of the valve systems improved the outcomes of the patients over the years and reduced complication rates (4–8). As a result, using TAVI to treat intermediate- and lower-risk patients with longer life expectancy is continuously expanding (4, 7, 9–14). The increasing lifespan of patients who underwent TAVI, combined with the desire to minimize recurrence of symptoms and the need for reintervention, had placed valve durability at the focus of attention (15–17). The data from randomized controlled trials (RCTs) examining the comparison of valve durability between TAVI and surgical aortic valve replacement (SAVR) were limited, and only one network meta-analysis had examined this issue (18). This meta-analysis focused on the comparison between the two most common TAVI valve systems, such as balloon-expandable valve and self-expanding valve, and compared each of these with SAVR without comparing TAVI with SAVR (18). In addition, since its publication, important new data have been published that were not included in the previous analysis (11, 19–22). Therefore, we aimed to conduct an updated meta-analysis comparing the durability between TAVI and SAVR using all data currently available from RCTs.

## Methods

The study protocol was written by TL and AL. We conducted a comprehensive search to identify studies in PubMed, Embase, and Cochrane CENTRAL, until December 2022, using a combination of keywords and MeSH terms for transcatheter aortic valve replacement, transcatheter aortic valve implantation, and surgical aortic valve implantation. References of all included trials and reviews identified were scanned for additional studies. All titles and abstracts were screened, and those thought to possibly meet the inclusion criteria were screened for eligibility using full text. The primary outcome was structural valve deterioration (SVD) that is defined as an intrinsic and permanent structural valvular change, causing AS or transvalvular aortic regurgitation (AR) (23, 24). Variability in definitions and criteria were found, and the data used in the pooled analysis were based on the criteria used in the individual studies included. Secondary outcomes were bioprosthetic valve failure (BVF; valve-related death, reintervention, or severe hemodynamic SVD), reintervention, aortic valve effective orifice area (EOA), mean pressure gradient (MG), and moderate–severe AR (transvalvular and/or paravalvular) (23, 24). We included studies with a follow-up period longer than 1 year. The longest follow-up data available were used in the pooled analysis.

Two reviewers (TL and AL) independently extracted the data and resolved conflicts by discussion. In studies where the data of interest were not available, we contacted the authors of the article with a request to share the information. In cases of data presented as figures without numerical data, extraction was done using Plot Digitizer software (PlotDigitizer, 3.1.5, 2023, https://plotdigitizer.com) as recommended by the Cochrane Collaboration.

Two authors (TL and AL) assessed the risk of bias. Cochrane's handbook tool was used to assess the studies (25). A funnel plot was used to assess publication bias. The systematic review and meta-analysis were conducted in compliance with the Cochrane Collaboration and Preferred Reporting Items for Systematic Reviews and Meta-Analyses statement (25). The meta-analysis was conducted using ReviewManager (RevMan) (Version 5.4. Copenhagen: The Nordic Cochrane Centre, The Cochrane Collaboration, 2020).

Heterogeneity between the included trials was assessed using the chi-squared test, and the *I*^2^ test was used to assess inconsistency. We used a fixed-effect model with Mantel–Haenszel method for pooling trial results throughout the review unless statistically significant heterogeneity was found (*P* < 0.10 or *I*^2^ > 50%), in which case we chose a random-effects model and used the inverse variance method. The values reported were two-tailed, and the hypothesis-testing results were considered significant at *P* < 0.05.

A sensitivity analysis was carried out by examining the effect of the exclusion of each study on the pooled results (“leave-one-out” analysis). The study was pre-registered in PROSPERO with ID number CRD42022363060.

## Results

**Figure 1:**
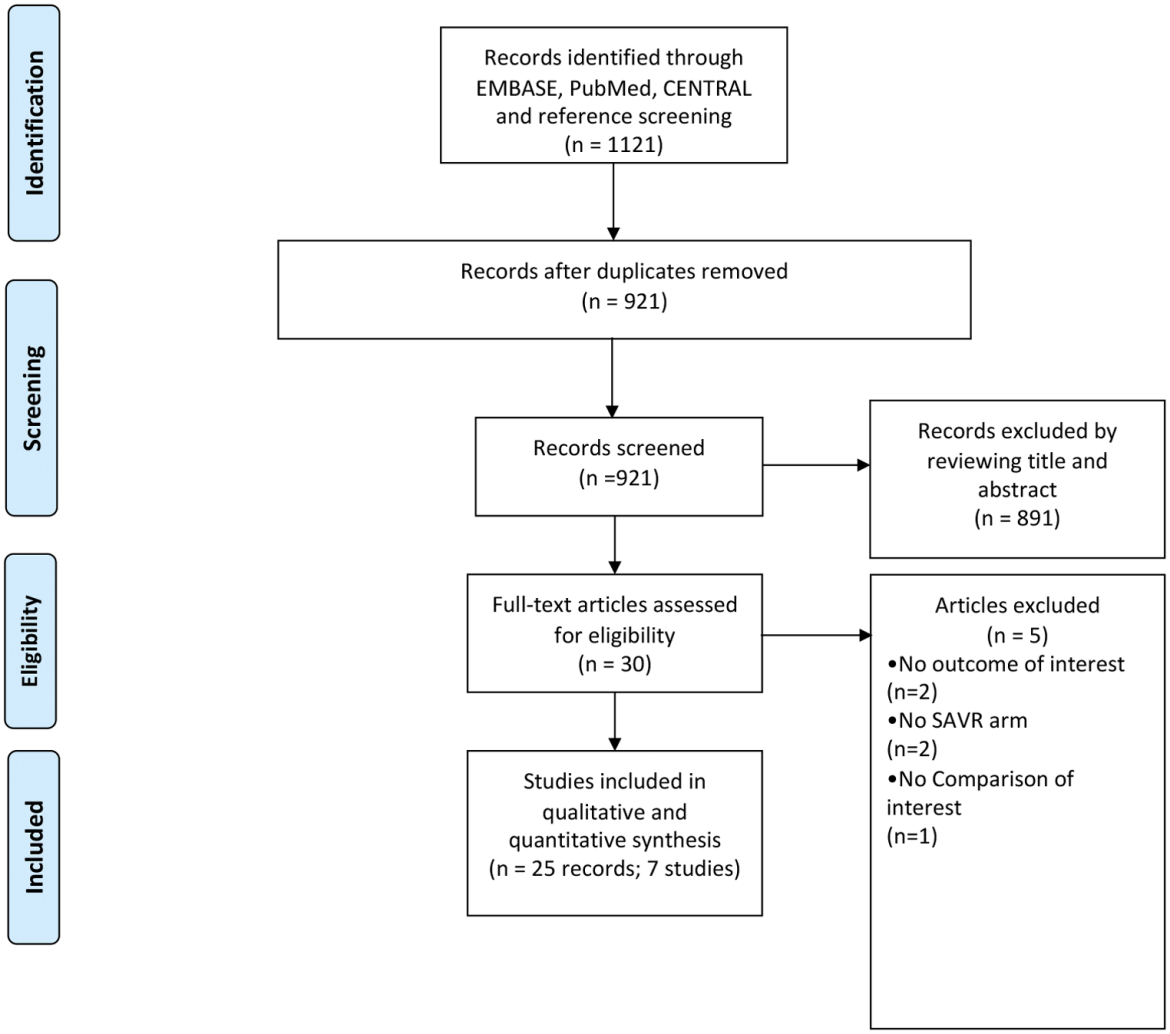
Study selection process for inclusion in the meta-analysis (PRISMA flow diagram).

A flow chart representing the study selection process is illustrated in Figure 1. Our initial search yielded 921 citations, 30 of which were judged to be potentially eligible and underwent a full-text review. Twenty-five publications from seven RCTs were found to be eligible for inclusion after the full-text review (4–7, 9, 11, 19–22, 26–40). Overall, our meta-analysis included data on 7,970 patients: 4,007 treated by TAVI and 3,963 treated by SAVR. Their mean age was 79%, and 59% were males. The longest follow-up available per study ranged from 2 to 8 years. The characteristics of the studies and patients included in this meta-analysis are presented in Table 1.

**Table 1:** Characteristics of studies and patients included in the meta-analysis.

Study	Year	Sample size	Follow-up	TAVI valve	STS	EuroScore	Age	Male	Risk of bias
Partner 1	2011	TAVI 348	5 years	Sapien	11.8 ± 3.3	29.3 ± 16.5^a^	83.6 ± 6.8	58%	Low
		SAVR 351			11.7 ± 3.5	29.2 ± 15.6^a^	84.5 ± 6.4	57%	
Partner 2	2016	TAVI 1011	5 years	Sapien XT	5.8 ± 2.1	—	81.5 ± 6.7	54%	Low
		SAVR 1021			5.8 ± 1.9	—	81.7 ± 6.7	55%	
Partner 3	2019	TAVI 496	2 years	Sapien 3	1.9 ± 0.7	1.5 ± 1.2	73.3 ± 5.8	68%	Low
		SAVR 454			1.9 ± 0.6	1.5 ± 0.9	73.6 ± 6.1	71%	
US CoreValve	2014	TAVI 394	5 years	CoreValve	7.3 ± 3.0	17.6 ± 13.0^a^	83.2 ± 7.1	54%	Some concerns
		SAVR 401			7.5 ± 3.2	18.4 ± 12.8^a^	83.5 ± 6.3	47%	
NOTION	2015	TAVI 145	8 years	CoreValve	2.9 ± 1.6	1.9 ± 1.2	79.2 ± 4.9	54%	Low
		SAVR 135			3.1 ± 1.7	2.0 ± 1.3	79.0 ± 4.7	53%	
SURTAVI	2017	TAVI 879	5 years	CoreValve/Evolut R	4.4 ± 1.5	11.9 ± 7.6^a^	79.9 ± 6.2	58%	Low
		SAVR 867			4.5 ± 1.6	11.6 ± 8.0^a^	79.8 ± 6.0	56%	
Evolut R	2019	TAVI 734	2 years	CoreValve/Evolut R/PRO	1.9 ± 0.7	—	74.0 ± 5.9	64%	Low
		SAVR 734			1.9 ± 0.7	—	73.8 ± 6.0	66%	

^a^ EuroScore, in other studies EuroScore II is reported. Year refers to the first study published.

STS, Society of Thoracic Surgeons.

### Structural valve deterioration

**Figure 2:**
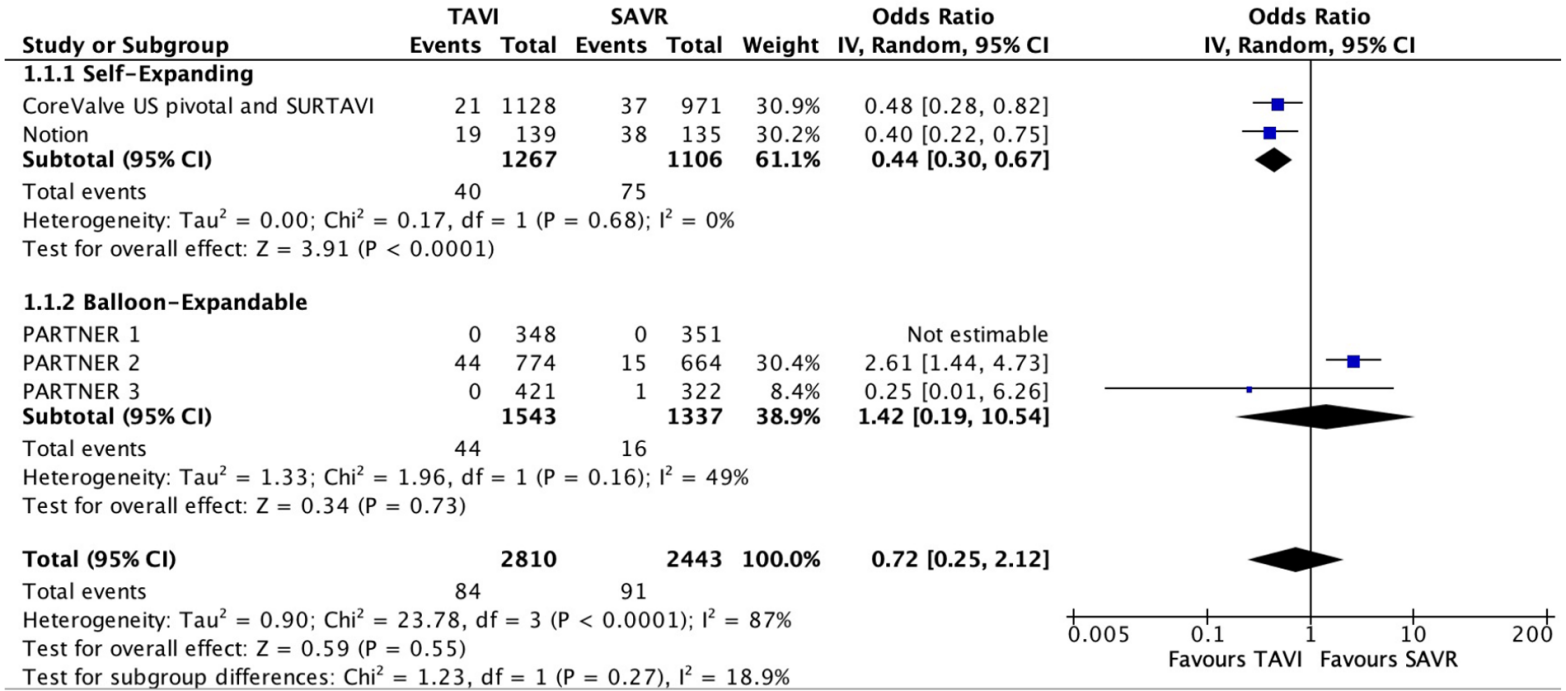
Forest plots for structural valve deterioration.

Five studies reported data with regard to SVD. Their follow-up ranged from 1 to 8 years. A total of 84 patients out of 2,810 (3.0%) were enrolled in the TAVI group, and 91 out of 2,443 (3.7%) in the SAVR group. Overall, no significant difference were found between the two groups (OR 0.72; 95% CI: 0.25–2.12; *P* = 0.55, *I*^2^ = 87%; Figure 2). However, in a subgroup analysis, a statistically significant lower risk was observed among patients who underwent self-expanding TAVI compared with SAVR (OR 0.44; 95% CI: 0.30–0.67; *P* < 0.01, *I*^2^ = 0%).

### Secondary outcomes

Only two studies reported data with regard to BVF. No statically significant difference was noted between the two groups (OR 1.50; 95% CI: 0.46–4.90); *P* = 0.5, *I*^2^ = 77%).

**Figure 3:**
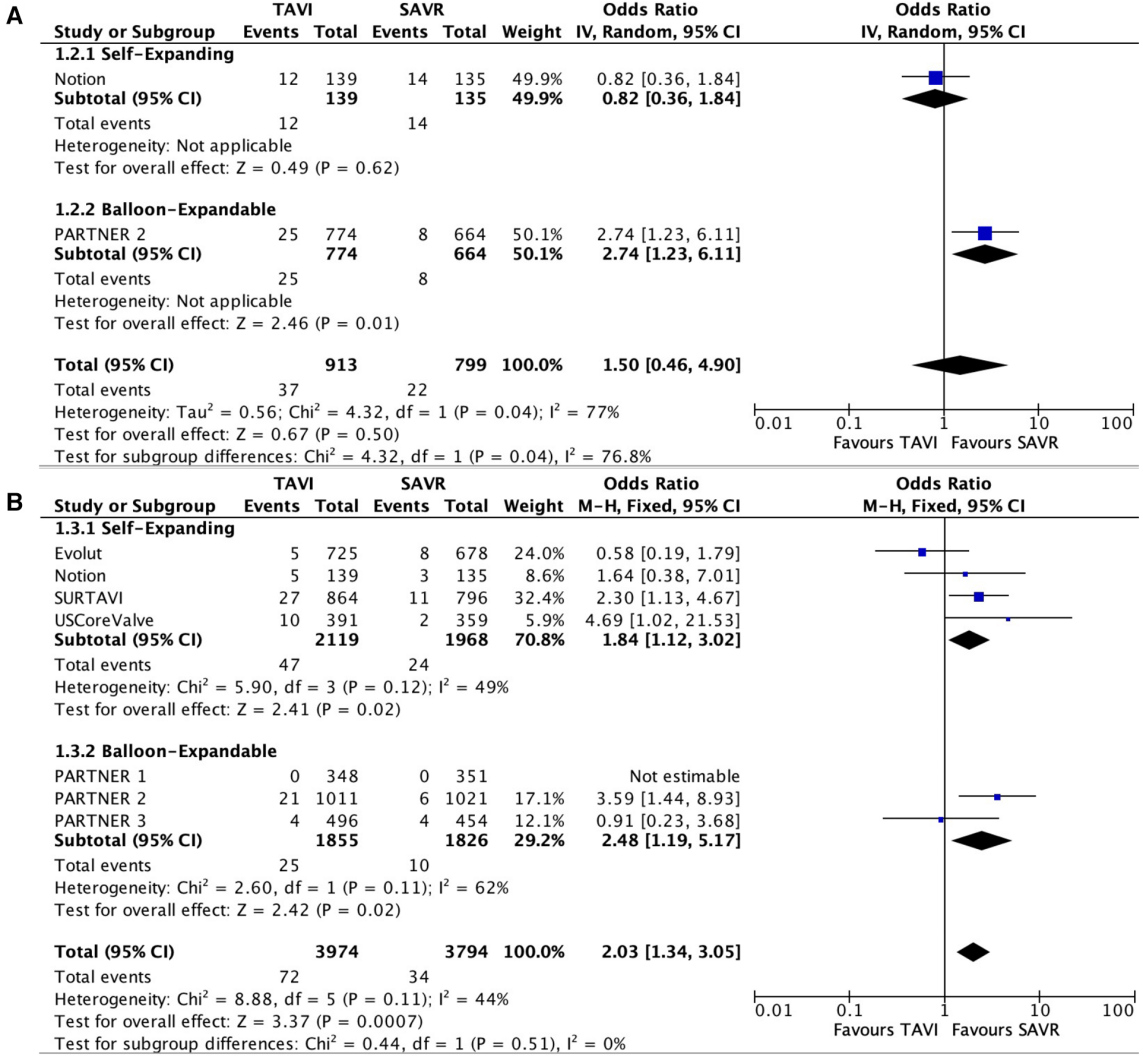
Forest plots for bioprosthetic valve failure (**A**) and reintervention (**B**).

Seven studies reported data with regard to reintervention. A statistically significant higher risk was noted for reintervention in the TAVI group compared with SAVR (OR 2.03; 95% CI: 1.34–3.05); *P* < 0.01, *I*^2^ = 44%). BVF and reintervention results are presented in Figure 3.

All RCTs provided data with regard to valve performances with follow-up ranges of 1–8 years. No significant difference was seen in mean pressure gradients between the two groups [(mean difference (MD) −1.61; 95% CI: −3.5 to 0.28); *P* = 0.1, *I*^2^ = 95%)]. Importantly, a significant heterogeneity was observed between the self-expanding subgroup and the balloon-expandable subgroup (*I*^2^ = 97%, *P* < 0.01). In the self-expanding group, the mean gradient was significantly lower than in the SAVR group (MD −3.25; 95% CI: −4.23 to −2.28; *P* < 0.01, *I*^2^ = 38%). In the balloon-expandable group, a statistically non-significant trend toward a higher mean gradient was reported in the TAVI group compared with the SAVR group (MD 1; 95% CI: −0.06 to 2.05; *P* = 0.06, *I*^2^ = 71%).

EOA was higher in the TAVI group than in the SAVR group (MD 0.20; 95% CI: 0.08–0.31); *P* < 0.01, *I*^2^ = 89%). This difference was mainly driven by the self-expanding subgroup (MD 0.3; 95% CI: 0.21–0.4; *P* < 0.01, *I*^2^ = 38%).

**Figure 4:**
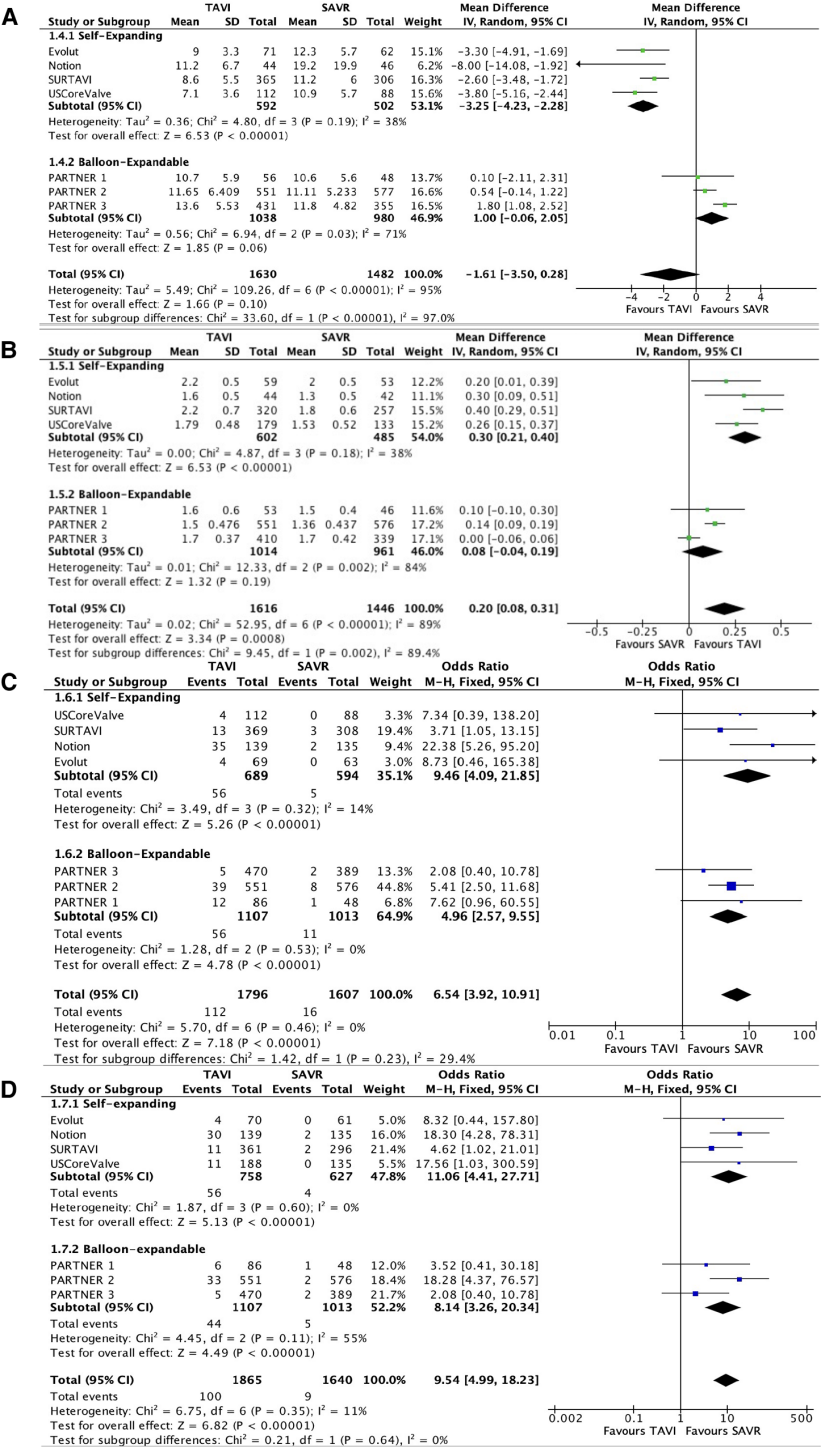
Forest plots for mean gradients (**A**), effective orifice area (**B**), moderate–severe aortic regurgitation (**C**), and moderate–severe paravalvular leak (**D**).

**Figure 5:**
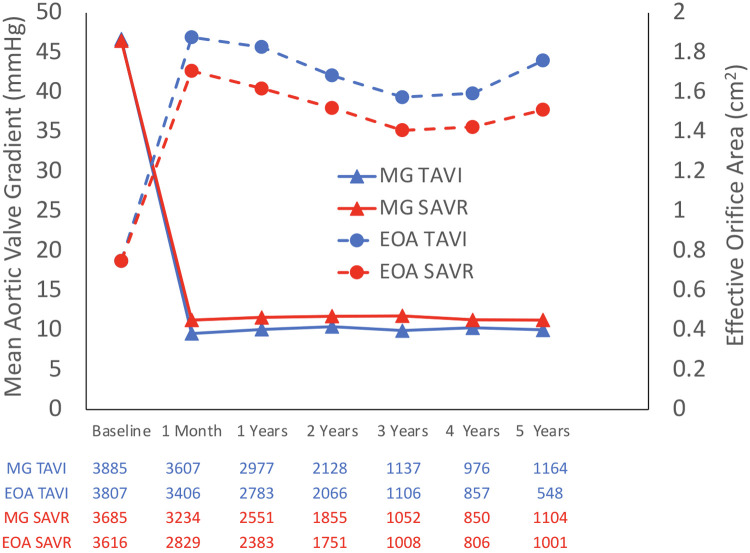
Mean pressure gradient and effective orifice area during a 5-year follow-up.

The rate of moderate–severe AR was higher in the TAVI group than that in the SAVR group, 112 patients out of 1,796 (6.2%) vs. 16 patients out of 1,607 (1%), respectively (OR 6.54; 95% CI: 3.92–10.91; *P* < 0.01, *I*^2^ = 0%), which was mainly driven by paravalvular leak (PVL). Valve performances are presented in Figure 4. MG and EOA during a 5-year follow-up period are presented in Figure 5.

### Sensitivity analysis

With the exclusion of the PARTNER 2 study, the SVD rate was reported to be significantly lower in the TAVI group (OR 0.44; 95% CI: 0.29–0.66; *P* < 0.01, *I*^2^ = 0%). The exclusion of PARTNER 1 study that used different SVD criteria did not change the statistical significance of the analysis.

After the exclusion of the PARTNER 3 study, the mean gradient was found to be significantly lower in the TAVI group (MD −2.24; 95% CI: −4.18 to −0.31; *P* = 0.02, *I*^2^ = 92%).

Exclusion of all other studies did not affect the statistical significance of the analysis.

## Discussion

We conducted the systematic review and meta-analysis including all RCTs that compared the valve durability and hemodynamic performances between TAVI and SAVR for a follow-up period longer than 1 year. We decided to use SVD as the primary outcome in our meta-analysis. SVD is one of the proposed categories of standardized definition of valve dysfunction, i.e., valvular changes causing AS or transvalvular AR. This contrasts with non-structural valve dysfunction (PVL, patient prosthesis mismatch), endocarditis, and valve thrombosis which are not caused by intrinsic valvular dysfunction (23, 24). Since the publication of the previous network meta-analysis examining valve durability, two important studies had been published comparing the SVD rate between TAVI and SAVR. The first study was an 8-year follow-up study of the Nordic Aortic Valve Intervention Trial (NOTION) which showed a lower risk of SVD in patients treated with self-expanding TAVI (21). The second study was a *post hoc* analysis of 5-year pooled data from two RCTs: US High-Risk Pivotal and SURTAVI trials (20). This study enrolled 4,762 patients and found a lower SVD rate among the self-expanding TAVI group compared with SAVR group. Importantly, this large-scale study found an association between SVD and all-cause mortality as well as cardiovascular mortality and therefore emphasized the clinical importance of this outcome as a surrogate marker for a worse prognosis of the patients. Interestingly, the advantage of TAVI was most pronounced in smaller annuli, and therefore further study of patient-level meta-analysis examining this aspect was suggested. In addition, it was well established that SVD rate was affected by the age of the patient, patient–prosthesis mismatch, and the use of lipid-lowering agent further emphasizing the need for analyses using patient-level data (41–43). It was worth mentioning that the studies included in the analysis used different criteria for SVD. However, these criteria generally overlapped, and therefore a pooled analysis was feasible. One exception was the PARTNER 1 study. However, excluding this study in a “leave-one-out” scrutiny did not significantly change the results. Another durability outcome we examined was BVF which was not reported in the previously published meta-analysis (18). This outcome was only reported in three studies: the NOTION study, PARTNER 2 study, and PARTNER 3 study (11, 21, 39). However, the latter did not report raw data with regard to the event rate and was therefore not included in our analysis. The two studies included used different, yet, overlapping BVF criteria, the EAPCI/ESC/EACT and the VARC-3 criteria, respectively. According to the pooled analysis, the rate of BVF was also comparable between TAVI and SAVR. However, due to a limited number of studies examining this outcome, it should be cautiously interpreted over a longer period of follow-up. In addition, it should be noted that in the PARTNER 2 study a higher rate of BVF was demonstrated in the TAVI group using the Sapien XT percutaneous valve that was not in clinical use anymore. Therefore, additional studies examining this outcome using contemporary devices are necessary (39).

In line with the previous network meta-analysis published by Ueyama et al., our meta-analysis found a favorable forward-flow hemodynamic profile of TAVI compared with SAVR with stable valve performances during the long-term follow-up (18). The hemodynamic advantage of TAVI was most prominently demonstrated in the self-expanding subgroup perhaps owing to their supra-annular functional design which facilitates larger effective valve diameter. This finding was consistent with a recently published meta-analysis from our group, comparing the latest-generation self-expanding valve with the balloon-expandable valve (44). Despite the favorable forward hemodynamic profile of TAVI, a significant inferiority was demonstrated with regard to the rate of moderate–severe AR and the rate of reintervention. Our pooled analysis was related to a total number of moderate–severe AR (i.e., transvalvular and PVL), and the higher rate of significant AR was mainly driven by PVL without a significant difference between the valve-type subgroups. This explains the fact that despite the higher rate of significant AR the rate of SVD was comparable between the two study arms since PVL is considered as non-SVD valve dysfunction. Since SVD and PVL are the two most common indications for reintervention, one can assume that the higher rate of PVL encountered in the TAVI group led to a higher reintervention rate (45). It has to be acknowledged that the characteristics of the surgical population were not always advantageous for optimized SAVR durability. For instance, in NOTION, 34% of patients received a valve prosthesis with lower durability performance (24% Saint Jude Trifecta, 10% Sorin Mitroflow). Despite the well-known negative effect of patient–prosthesis mismatch on valve durability, SAVR patients in PARTNER 3 received a labeled prosthesis of ≤21 mm in 20% of patients (9, 40, 46, 47). The advantage of SAVR with regard to reintervention should be emphasized in light of these considerations. The higher reintervention rate in the TAVI group was in line with a recently published meta-analysis (48).

It has been previously established that balloon-expandable and self-expanding valves have a different efficacy and safety profile. This is reflected by differences in pacemaker implantation rate, periprocedural bleeding rate, and hemodynamic profile (44) Combined with the results of the current study examining the durability of TAVI valves, we believe that a set of considerations are obtained that should be incorporated into the heart team decision-making (44).

Our meta-analysis has several strengths. First, our analysis has the longest follow-up period available today of up to 8 years. In addition, this is the most comprehensive analysis conducted including five follow-up studies that were not included in the previous meta-analysis published (11, 18–22). In addition, the pooled analysis included only data from RCTs without data from observational studies, therefore reflecting high-quality data and reliable analysis. Yet, several limitations should be mentioned. First, the patients included in the RCTs were at a wide range of surgical risk, and it is possible that the final study population in the meta-analysis does not reflect the real-world population. However, since we mainly tested valvular outcomes and not “hard outcomes,” the effect is expected to be minimal; yet, long-term real-life registries data are needed to examine valve durability and confirm our results. Second, follow-up echocardiography was not available for a significant number of patients. The impact of selective echocardiography on results is unpredictable. Sicker patients with poorer valve performance could have been underrepresented if they were unable to complete the follow-up. On the other hand, patients feeling well, with a lower rate of valve degeneration, may also feel disinclined to continue the follow-up, leading to their underrepresentation. Third, older-generation valves were used in some studies and may not reflect modern-era TAVI. Fourth, we did not have access to individual patient data, and the analysis is based on aggregate data and therefore should be interpreted with caution. Fifth, in some of the analyses, a significant heterogeneity was demonstrated between the studies.

Lastly, although only RCTs were included in the analysis, few biases should be considered. In RCTs comparing TAVI with SAVR, systematic imbalances in the proportion of deviation from randomly assigned treatment (DAT), high loss to follow-up (8.9% at 5 years), and receipt of additional procedures and additional myocardial revascularization that can pose a serious threat to internal validity due to a high risk of performance and attrition biases were noted (49).

## Conclusions

In conclusion, according to our analysis, TAVI provided a comparable risk of SVD with favorable hemodynamic profile compared with SAVR. However, a higher rate of significant AR and reintervention was demonstrated. These results provide an important insight regarding valve durability that should be considered when tailoring treatment to the individual patient. Additional real-life and expanded data over a longer period of time are needed to confirm our results.

## Data Availability

The original contributions presented in the study are included in the article/**Supplementary Material**, further inquiries can be directed to the corresponding author.
