# Supplementary material for: Comparison of middle-term valve durability between transcatheter aortic valve implantation and surgical aortic valve replacement: an updated systematic review and meta-analysis of RCTs
Source: Front Cardiovasc Med. 2023 Sep 13;10:1242608. doi: 10.3389/fcvm.2023.1242608 (PMC10525352; doi:10.3389/fcvm.2023.1242608)
Supplement: Supplementary file 1 [file Datasheet1.docx]

**Supplemental material**

Intended for publication as an online data supplement

**Table S1.** SVD and BVF criteria

| Study | Definition |
| --- | --- |
| Partner 1 | **Structural valve deterioration**  Structural valve deterioration requiring surgical valve replacement in either group. |
| Partner 2 | **Structural valve deterioration**  Intrinsic permanent changes to the prosthetic valve, including leaflet tear, disruption, flail leaflet, leaflet fibrosis and/or calcification.  **Bioprosthetic valve failure**  **•** Stage 1: Any significant bioprosthetic valve dysfunction with clinically expressive criteria (new-onset or worsening symptoms, LV dilation/ hypertrophy/ dysfunction, or pulmonary hypertension) OR Stage 3 hemodynamic valve deterioration related to permanent changes to the prosthetic valve.  • Stage 2: Aortic valve reoperation or reintervention.  • Stage 3: Valve-related death. |
| Partner 3 | Hemodynamic valve deterioration defined as increase in mean gradient ≥10 mm Hg with concomitant decrease in AVA ≥0.3 cm2 or in DVI ≥0.1 or a new onset or worsening of transvalvular AR by at least 1 class (3-class scheme) between 30 days and 1 year with ≥ moderate AR at 1 year |
| US CoreValve and SURTAVI | **Structural valve deterioration**  Moderate or greater hemodynamic SVD.  Moderate SVD was defined as (1) hemodynamic valve deterioration (HVD) showing an increase in mean aortic gradient of 10 mm Hg or greater from discharge or 30-day echocardiography to last available echocardiography with a final mean gradient of 20mm Hg or greater or (2) new occurrence or increase of 1 grade or more of intraprosthetic AR resulting in moderate or severe AR. Severe SVD was defined as (1) HVD showing an increase in mean gradient of 20 mm Hg or greater from discharge or 30-day echocardiography to last available echocardiography with a final mean gradient of 30 mm Hg or greater or (2) new occurrence or increase of 2 grades or more of intraprosthetic AR resulting in severe |
| NOTION | **Structural valve deterioration**  Moderate-Severe SVD according to EAPCI/ESC/EACTS criteria  **Bioprosthetic valve failure**  one of the following three criteria: (i) valve-related death (death caused by bioprosthetic valve deterioration (SVD and non-SVD; BVD * or sudden unexplained death following diagnosis of BVD); (ii) severe hemodynamic SVD; and (iii) aortic valve re-intervention following diagnosis of BVD. |

**BVD -* *Bioprosthetic valve dysfunction was categorized into four groups: (i) structural valve deterioration (SVD) defined as moderate SVD (mean transvalvular gradient >_20 mmHg, increase in mean gradient >_10 mmHg from 3 months post-procedure, or new or worsening moderate intraprosthetic aortic regurgitation from 3 months post-procedure) and severe SVD (mean transvalvular gradient >_40 mmHg, increase in mean gradient >_20 mmHg from 3 months post-procedure, or new or worsening severe intra-prosthetic aortic regurgitation from 3 months post-procedure); (ii) non-structural valve deterioration (NSVD) defined as moderate to severe patient-prosthesis mismatch (PPM (indexed effective orifice area <_0.85 cm2/m2 for moderate PPM and <_0.65 cm2/m2 for severe PPM) at 3 months, or more than mild paravalvular leakage (PVL); (iii) bioprosthetic valve thrombosis defined as thrombus development on any structure of the prosthetic valve leading to dysfunction; and (iv) infectious endocarditis diagnosed according to the modified Duke criteria.*

**Search strategy**

**Pubmed (296 publications)**

(“TAVI”[All Fields] OR “TAVR”[All Fields] OR "Transcatheter Aortic Valve Implantation"[All Fields] OR "Transcatheter Aortic Valve replacement"[All Fields] OR "Transcatheter Aortic Valve Replacement"[Mesh] OR “transcatheter”[All Fields])

AND (“SAVR”[All Fields] OR "surgical Aortic Valve replacement"[All Fields] OR "surgical Aortic Valve implantation"[All Fields] OR “AVR”[All Fields] OR "Aortic Valve Replacement"[All Fields])

**Embase (320 publications)**

('transcatheter aortic valve implantation'/exp OR 'transcatheter aortic valve implantation' OR 'transcatheter aortic valve replacement'/exp OR 'transcatheter aortic valve replacement' OR 'tavi'/exp OR 'tavi' OR 'tavr' OR 'transcatheter') AND ('surgical aortic valve replacement'/exp OR 'surgical aortic valve replacement' OR 'surgical aortic valve implantation')

**CENTRAL (504 publications)**

#1 transcatheter aortic valve implantation

#2 transcatheter aortic valve replacement

#3 MeSH descriptor: [Transcatheter Aortic Valve Replacement] 2 tree(s) exploded

#4 surgical aortic valve implantation

#5 surgical aortic valve replacement

#6 #1 OR #2 OR #3

#7 #4 OR #5

#8 #6 AND #7 in Trials
